# Antimicrobial activity of ceftazidime-avibactam and comparators against levofloxacin-resistant *Escherichia coli* collected from four geographic regions, 2012–2018

**DOI:** 10.1186/s12941-022-00504-8

**Published:** 2022-03-21

**Authors:** Gregory G. Stone, Meredith A. Hackel

**Affiliations:** 1 Hospital Business Unit, Global Products Development, Groton Laboratories, 558 Eastern Point Road, Groton, CT 06340 USA; 2 IHMA, Schaumburg, IL USA

**Keywords:** Ceftazidime-avibactam, Levofloxacin-resistant *Escherichia coli*, Extended-spectrum β-lactamase

## Abstract

**Background:**

Increases in resistance to fluoroquinolones have been correlated with the use of levofloxacin in the treatment of infections caused by *Escherichia coli.* The analysis presents the in vitro activity of ceftazidime-avibactam and comparator agents against 10,840 levofloxacin-resistant *E. coli* isolates collected from four geographic regions (Africa/Middle East, Europe, Asia/South Pacific, Latin America) between 2012 and 2018.

**Methods:**

Non-duplicate clinical isolates of *E. coli* were collected from participating centres and shipped to IHMA, Inc., (Schaumburg, IL, USA). Susceptibility testing was performed with frozen broth microdilution panels manufactured by IHMA, according to CLSI guidelines. Levofloxacin-resistance was defined at a minimum inhibitory concentration of ≥ 2 mg/L. Isolates collected between 2012 and 2015 were tested for extended-spectrum β-lactamase (ESBL) activity by determining susceptibility to cefotaxime, cefotaxime-clavulanate, ceftazidime, and ceftazidime-clavulanate as recommended by CLSI guidelines. Isolates collected between 2016 and 2018 were identified as ESBL-positive by genotype using multiplex polymerase chain reaction assays.

**Results:**

A total of 74.8% of levofloxacin-resistant *E. coli* isolates in the analysis were from three culture sources: urinary tract infections (N = 3229; 29.8%), skin and skin structure infections (N = 2564; 23.7%) and intra-abdominal infections (N = 2313; 21.3%). Susceptibility rates to ceftazidime-avibactam were consistently high in all regions against both ESBL-positive (97.0% in Asia/South Pacific to 99.7% in Africa/Middle East and Latin America) and ESBL-negative isolates (99.4% in Asia/South Pacific to 100% in Latin America). Susceptibility was also high in each region among ESBL-positive and ESBL-negative isolates to colistin (≥ 98.5%), imipenem (≥ 96.5%), meropenem (≥ 96.5%) and tigecycline (≥ 94.1%).

**Conclusions:**

Antimicrobial susceptibility to ceftazidime-avibactam among levofloxacin-resistant *E. coli* isolates, including ESBL-positive isolates, collected from four geographical regions between 2012 and 2018 was consistently high. Susceptibility to the comparator agents colistin, tigecycline, imipenem and meropenem was also high.

**Supplementary Information:**

The online version contains supplementary material available at 10.1186/s12941-022-00504-8.

## Background

The frequency of antimicrobial resistance has increased worldwide and has been associated with the inappropriate use of antimicrobials [1]. Fluoroquinolones, which have high bioavailability, oral administration and good tissue distribution [2], are commonly used agents; however, increases in resistance to fluoroquinolones have been correlated with the use of levofloxacin in the treatment of infections caused by *E. coli* [3]. Resistance to fluoroquinolones often arises by mutations in the drug targets, DNA gyrase and DNA topoisomerase IV [4, 5], and a frequency of > 20% resistance to fluoroquinolones among uropathogens, including *E. coli* has been reported [6, 7]. The availability of treatment options is further complicated in infections caused by members of the Enterobacterales as resistance to fluoroquinolones has been associated with extended-spectrum β-lactamase (ESBL) positive isolates, and ESBL production is associated with the hydrolysis of penicillins and β-lactams, including third-generation cephalosporins [8–10]. ESBL-producing bacteria are now pervasive worldwide, and according to one estimate, over 1.5 billion people are colonised with ESBL-producing Enterobacterales [11].

Avibactam is a diazabicyclooctane, non-β-lactam, β-lactamase-inhibitor, and the combination of ceftazidime with avibactam possesses in vitro activity against Enterobacterales carrying β-lactamases of Ambler class A (ESBLs and *Klebsiella pneumoniae* carbapenemases), class C (AmpC cephalosporinases) and some class D (e.g. OXA-48-type, many of which co-carry ESBLs) [12–16]. The in vitro activity of ceftazidime-avibactam and a panel of comparator agents has been tracked via the International Network for Optimal Resistance Monitoring (INFORM) surveillance program, which was established in 2012, and the Antimicrobial Testing Leadership and Surveillance (ATLAS) study [17], which succeeded INFORM.

The data presented here describe the in vitro activity of ceftazidime-avibactam and comparator agents against ESBL-positive and ESBL-negative levofloxacin-resistant *E. coli* isolates collected from four geographic regions (Africa/Middle East, Europe, Asia/South Pacific, Latin America) between 2012 and 2018.

## Materials and methods

### Bacterial isolates

Non-duplicate clinical isolates of *E. coli* were collected from participating centres in Africa/Middle East, Asia/South Pacific, Europe and Latin America between 2012 and 2018. All isolates were obtained from specimens collected from patients with community-associated or hospital-associated infections from intra-abdominal, skin, wounds, blood, respiratory tract, urine (limited to no more than 25% of all isolates), fluids, and other defined sources. Each site was requested to collect 25 *E. coli* isolates, and only one isolate per patient were accepted according to the protocol. All isolates were determined to be clinically significant by participating laboratory algorithms and were collected irrespective of antimicrobial susceptibility profile. Following their shipment to the central reference laboratory (IHMA, Schaumburg, IL, USA), samples were identified using matrix-assisted laser desorption ionization-time of flight (MALDI-TOF) mass spectrometry (Bruker Biotyper, Bruker Daltonics, Billerica, MA, USA).

### Susceptibility testing

Susceptibility testing was performed according to CLSI and ISO guidelines, [18, 19] with frozen broth microdilution panels manufactured by IHMA. Panel preparation and quality control followed guidelines from the CLSI [18, 20]. Avibactam was tested at a fixed concentration of 4 mg/L in combination with doubling dilutions of ceftazidime (testing range, ≤ 0.015–128 mg/L). MICs were interpreted using EUCAST 2020 breakpoints, version 10.0 [21] Resistance to levofloxacin was based on EUCAST guidelines and was defined as a minimum inhibitory concentration (MIC) of ≥ 2 mg/L. Isolates collected between 2012 and 2015, with MICs of ≥ 2 mg/L to ceftazidime or aztreonam, were tested for ESBL activity by determining susceptibility to cefotaxime, cefotaxime-clavulanate, ceftazidime, and ceftazidime-clavulanate as recommended by CLSI guidelines [20]. Isolates collected between 2016 and 2018 with MICs ≥ 2 mg/L to meropenem, ceftazidime or aztreonam were screened for β-lactamase genes, using multiplex polymerase chain reaction assays, and ESBL-positive isolates were identified by genotype [22]. All detected β-lactamase genes, excluding original spectrum β-lactamases were amplified using flanking primers and sequenced. Sequences were compared against publicly available databases.

### Statistical analyses

The Cochran-Armitage Trend Test was used to assess changes over the study years in the proportion of levofloxacin-resistant *E. coli* isolates that were identified as ESBL-positive. A p-value of < 0.01 was interpreted as statistically significant as the n values in the analysis were high and therefore the test was likely to be over-powered. Analyses were performed with SAS® version 9.4 (SAS Institute Inc., Cary, NC, USA).

## Results

### Distribution of levofloxacin-resistant *E. coli* isolates

A total of 10,840 isolates collected from four geographic regions and identified as resistant to levofloxacin were included in the analysis. Isolates were most commonly collected from UTIs (N = 3229; 29.8%), followed by 2564 (23.7%) from skin and skin structure infections, 2313 (21.3%) from intra-abdominal infections, 1482 (13.7%) from lower respiratory tract infections, and 1204 (11.1%) from bloodstream infections, whilst 48 (0.4%) were from an unknown or other source. A similar distribution was observed among ESBL-positive and ESBL-negative isolates (data not shown).

The highest proportion of isolates were collected from Europe (N = 4663; 43.0%). The proportion of isolates collected from Latin America (N = 2699; 24.9%) and Asia/South Pacific (N = 2337; 21.6%) were similar, and a minority were from Africa/Middle East (N = 1141; 10.5%).

### Analysis of regions combined against levofloxacin-resistant *E. coli*

Table 1 shows the in vitro activity of ceftazidime-avibactam and comparators against levofloxacin-resistant ESBL-positive and ESBL-negative *E. coli* when data from all regions of collection were combined. Rates of susceptibility to ceftazidime-avibactam and colistin were similar (≥ 99.0%) in both sets of isolates. Other comparator agents with high susceptibility rates against both sets of isolates were meropenem and imipenem (≥ 98.5%), and tigecycline (≥ 94.6%). A high susceptibility rate was observed to amikacin among ESBL-negative isolates (95.1%); however, a lower rate of 83.4% was observed among ESBL-positive isolates. For cefepime, ceftazidime and aztreonam relatively high rates of susceptibility were observed among ESBL-negative isolates (≥ 88.5%); however, a susceptibility rate of < 10% was seen among ESBL-positive isolates.

**Table 1:** Activity of ceftazidime-avibactam and comparator agents against levofloxacin-resistant *E. coli*; ATLAS, 2012–2018

Antimicrobial	MIC_50_ (mg/L)	MIC_90_ (mg/L)	Range (mg/L)	%S	%I	%R
*ESBL-positive (N = 5749)*						
Ceftazidime-avibactam	0.12	0.5	≤ 0.015– ≥ 256	99.0	–	1.0
Ceftazidime^a^	32	128	0.12– ≥ 256	5.3	14.8	79.9
Cefepime^a^	32	32	≤ 0.12– ≥ 64	3.0	10.0	86.9
Ampicillin	≥ 64	≥ 64	1– ≥ 64	0.2	–	99.8
Amoxicillin-clavulanate^a^	16	32	≤ 0.12– ≥ 64	29.3	–	70.7
Piperacillin-tazobactam	8	64	≤ 0.25– ≥ 256	64.8	15.2	20.0
Aztreonam^a^	32	128	0.06– ≥ 256	0.2	8.2	91.6
Imipenem	0.25	0.25	≤ 0.03– ≥ 16	98.5	0.4	1.1
Meropenem	0.03	0.06	≤ 0.004– ≥ 32	98.5	0.6	1.0
Colistin^b^ (N = 4470)	0.25	1	≤ 0.06– ≥ 16	99.1	–	0.9
Amikacin	4	16	0.5– ≥ 128	83.4	–	16.6
Tigecycline	0.25	0.5	≤ 0.015– ≥ 16	95.7	–	4.3
*ESBL-negative (N = 5091)*						
Ceftazidime-avibactam	0.12	0.25	≤ 0.015– ≥ 256	99.6	–	0.4
Ceftazidime	0.25	4	≤ 0.015– ≥ 256	88.5	2.3	9.2
Cefepime	≤ 0.12	1	≤ 0.12– ≥ 64	91.8	3.8	4.4
Ampicillin	≥ 64	≥ 64	≤ 0.5– ≥ 64	17.4	–	82.6
Amoxicillin-clavulanate	8	32	≤ 0.12– ≥ 64	51.4	–	48.6
Piperacillin-tazobactam	2	64	≤ 0.12– ≥ 256	81.9	5.0	13.1
Aztreonam	0.12	4	≤ 0.015– ≥ 256	88.9	2.7	8.3
Imipenem	0.12	0.25	≤ 0.03– ≥ 16	99.1	0.3	0.6
Meropenem	0.03	0.06	≤ 0.004– ≥ 32	99.3	0.3	0.4
Colistin^b^ (N = 3864)	0.25	1	≤ 0.06– ≥ 16	99.0	–	1.0
Amikacin	2	8	≤ 0.25– ≥ 128	95.1	–	4.9
Tigecycline	0.25	0.5	≤ 0.015–4	94.6	–	5.4

– Indicates no breakpoint for the agent

*ESBL* extended-spectrum β-lactamase, *%I* percentage of isolates susceptible, increased exposure, *MIC* minimum inhibitory concentration, *MIC*_*50*_ MIC required to inhibit growth of 50% of isolates (mg/L), *MIC*_*90*_ MIC required to inhibit growth of 90% of isolates (mg/L), *%R* percentage of isolates resistant, *%S* percentage of isolates susceptible, standard dosing

^a^Not suitable for use in the treatment of infections caused by ESBL-positive isolates

^b^Colistin was included on the comparator panel from 2014 onwards

### Analysis by region against levofloxacin-resistant *E. coli*

For the regional analysis of all years pooled (2012–2018), presented in Table 2, susceptibility rates to ceftazidime-avibactam were consistently high in all regions for both ESBL-positive (97.0% in Asia/South Pacific to 99.7% in Africa/Middle East and Latin America) and ESBL-negative (99.4% in Asia/South Pacific to 100% in Latin America) levofloxacin-resistant *E. coli*. High susceptibility rates were also observed in each region among ESBL-positive and ESBL-negative isolates for colistin (≥ 98.5%), imipenem (≥ 96.5%), meropenem (≥ 96.5%) and tigecycline (≥ 94.1%).

**Table 2:** Activity of ceftazidime-avibactam and comparator agents against levofloxacin-resistant *E. coli*; ATLAS, by region, 2012–2018

Antimicrobial	MIC_50_ (mg/L)	MIC_90_ (mg/L)	Range (mg/L)	%S	%I	%R
*Africa/Middle East, ESBL-positive (N = 609)*						
Ceftazidime-avibactam	0.25	0.5	≤ 0.015– ≥ 256	99.7	–	0.3
Ceftazidime^a^	32	128	0.12– ≥ 256	2.8	13.3	83.9
Cefepime^a^	32	≥ 64	0.25– ≥ 64	2.5	7.2	90.3
Ampicillin	≥ 64	≥ 64	16– ≥ 64	0.0	–	100
Amoxicillin-clavulanate^a^	16	32	1– ≥ 64	26.9	–	73.1
Piperacillin-tazobactam	8	128	≤ 0.25– ≥ 256	61.1	18.2	20.7
Aztreonam^a^	64	128	2– ≥ 256	0.0	5.3	94.7
Imipenem	0.25	0.25	0.06– ≥ 16	99.3	0.3	0.3
Meropenem	0.03	0.06	0.015– ≥ 32	99.5	0.3	0.2
Colistin^b^ (N = 472)	0.5	1	≤ 0.06–8	99.2	–	0.8
Amikacin	4	16	0.5– ≥ 128	85.2	–	14.8
Tigecycline	0.25	0.5	0.06–4	96.2	–	3.8
*Africa/Middle East, ESBL-negative (N = 532)*						
Ceftazidime-avibactam	0.12	0.25	≤ 0.015– ≥ 256	99.6	–	0.4
Ceftazidime	0.25	0.5	≤ 0.015– ≥ 256	93.0	1.3	5.6
Cefepime	≤ 0.12	1	≤ 0.12– ≥ 64	92.7	3.2	4.1
Ampicillin	≥ 64	≥ 64	1– ≥ 64	15.0	–	85.0
Amoxicillin-clavulanate	8	32	≤ 0.12– ≥ 64	56.0	–	44.0
Piperacillin-tazobactam	2	32	0.5– ≥ 256	82.3	5.6	12.0
Aztreonam	0.12	0.5	≤ 0.015– ≥ 256	93.0	1.5	5.5
Imipenem	0.12	0.25	0.06– ≥ 16	99.1	0.4	0.6
Meropenem	0.03	0.06	0.008–16	99.2	0.2	0.6
Colistin^b^ (N = 413)	0.25	1	≤ 0.06– ≥ 16	98.5	–	1.5
Amikacin	2	8	0.5– ≥ 128	94.4	–	5.6
Tigecycline	0.25	0.5	0.06–4	94.9	–	5.1
*Asia/South Pacific, ESBL-positive (N = 1283)*						
Ceftazidime-avibactam	0.12	0.5	≤ 0.015– ≥ 256	97.0	–	3.0
Ceftazidime^a^	16	128	0.25– ≥ 256	6.3	16.4	77.3
Cefepime^a^	32	≥ 64	≤ 0.12– ≥ 64	1.5	11.0	87.5
Ampicillin	≥ 64	≥ 64	4– ≥ 64	0.2	–	99.8
Amoxicillin-clavulanate^a^	16	32	2– ≥ 64	41.6	–	58.4
Piperacillin-tazobactam	4	128	0.5– ≥ 256	74.0	9.8	16.1
Aztreonam^a^	32	128	0.06– ≥ 256	0.5	7.9	91.7
Imipenem	0.25	0.5	≤ 0.03– ≥ 16	96.5	0.3	3.2
Meropenem	0.03	0.12	0.008– ≥ 32	96.5	0.5	3.0
Colistin^b^ (N = 1012)	0.25	1	≤ 0.06– ≥ 16	98.8	–	1.2
Amikacin	4	16	0.5– ≥ 128	89.8	–	10.2
Tigecycline	0.25	0.5	0.03– ≥ 16	94.3	–	5.7
*Asia/South Pacific, ESBL-negative (N = 1054)*						
Ceftazidime-avibactam	0.12	0.25	≤ 0.015– ≥ 256	99.4	–	0.6
Ceftazidime	0.25	32	≤ 0.015– ≥ 256	77.9	3.1	19.0
Cefepime	≤ 0.12	1	≤ 0.12– ≥ 64	91.4	3.9	4.7
Ampicillin	≥ 64	≥ 64	≤ 0.5– ≥ 64	17.0	–	83.0
Amoxicillin-clavulanate	8	32	≤ 0.12– ≥ 64	52.4	–	47.6
Piperacillin-tazobactam	2	32	0.25– ≥ 256	83.5	4.9	11.6
Aztreonam	0.12	16	≤ 0.015– ≥ 256	79.7	4.6	15.7
Imipenem	0.25	0.5	≤ 0.03– ≥ 16	99.3	0.2	0.5
Meropenem	0.03	0.06	≤ 0.004– ≥ 32	99.4	0.1	0.5
Colistin^b^ (N = 780)	0.25	1	≤ 0.06– ≥ 16	99.4	–	0.6
Amikacin	2	8	≤ 0.25– ≥ 128	96.5	–	3.5
Tigecycline	0.25	0.5	0.03–4	94.7	–	5.3
*Europe, ESBL-positive (N = 2290)*						
Ceftazidime-avibactam	0.12	0.5	≤ 0.015– ≥ 256	99.6	–	0.4
Ceftazidime^a^	16	128	0.12– ≥ 256	6.0	17.2	76.9
Cefepime^a^	32	≥ 64	≤ 0.12– ≥ 64	4.6	12.6	82.8
Ampicillin	≥ 64	≥ 64	1– ≥ 64	0.3	–	99.7
Amoxicillin-clavulanate^a^	16	32	≤ 0.12– ≥ 64	25.9	–	74.1
Piperacillin-tazobactam	8	128	≤ 0.25– ≥ 256	61.3	15.8	22.9
Aztreonam^a^	32	128	0.5– ≥ 256	0.3	10.3	89.4
Imipenem	0.25	0.25	≤ 0.03– ≥ 16	99.0	0.4	0.6
Meropenem	0.03	0.06	≤ 0.004– ≥ 32	98.9	0.6	0.5
Colistin^b^ (N = 1754)	0.5	1	≤ 0.06– ≥ 16	99.1	–	0.9
Amikacin	4	16	0.5– ≥ 128	79.9	–	20.1
Tigecycline	0.25	0.5	≤ 0.015–4	96.3	–	3.7
*Europe, ESBL-negative (N = 2373)*						
Ceftazidime-avibactam	0.12	0.25	≤ 0.015– ≥ 256	99.5	–	0.5
Ceftazidime	0.25	1	0.03– ≥ 256	91.0	2.4	6.7
Cefepime	≤ 0.12	1	≤ 0.12– ≥ 64	91.1	4.3	4.6
Ampicillin	≥ 64	≥ 64	≤ 0.5– ≥ 64	16.0	–	84.0
Amoxicillin-clavulanate	16	32	≤ 0.12– ≥ 64	47.8	–	52.2
Piperacillin-tazobactam	2	64	≤ 0.12– ≥ 256	79.6	5.1	15.3
Aztreonam	0.12	1	≤ 0.015– ≥ 256	91.4	2.6	6.0
Imipenem	0.12	0.25	≤ 0.03– ≥ 16	98.7	0.5	0.8
Meropenem	0.03	0.06	≤ 0.004– ≥ 32	99.2	0.4	0.4
Colistin^b^ (N = 1786)	0.25	1	≤ 0.06– ≥ 16	98.9	–	1.1
Amikacin	2	8	0.5– ≥ 128	95.0	–	5.0
Tigecycline	0.25	0.5	≤ 0.015–4	94.1	–	5.9
*Latin America, ESBL-positive (N = 1567)*						
Ceftazidime-avibactam	0.12	0.5	≤ 0.015– ≥ 256	99.7	–	0.3
Ceftazidime^a^	32	128	0.5– ≥ 256	4.3	10.7	84.9
Cefepime^a^	32	32	≤ 0.12– ≥ 64	2.3	6.6	91.1
Ampicillin	≥ 64	≥ 64	8– ≥ 64	0.1	–	99.9
Amoxicillin-clavulanate^a^	16	32	≤ 0.12– ≥ 64	25.3	–	74.7
Piperacillin-tazobactam	8	64	≤ 0.25– ≥ 256	63.8	17.7	18.5
Aztreonam^a^	64	128	0.5– ≥ 256	0.1	6.4	93.5
Imipenem	0.12	0.25	0.06– ≥ 16	99.2	0.4	0.4
Meropenem	0.03	0.06	0.015– ≥ 32	99.0	0.6	0.4
Colistin^b^ (N = 1232)	0.25	1	≤ 0.06–8	99.1	–	0.9
Amikacin	4	16	0.5– ≥ 128	82.6	–	17.4
Tigecycline	0.25	0.5	≤ 0.015–4	95.8	–	4.2
*Latin America, ESBL-negative (N = 1132)*						
Ceftazidime-avibactam	0.12	0.25	≤ 0.015–4	100	–	0.0
Ceftazidime	0.25	1	0.03– ≥ 256	91.1	1.7	7.2
Cefepime	≤ 0.12	1	≤ 0.12– ≥ 64	93.3	3.1	3.6
Ampicillin	≥ 64	≥ 64	1– ≥ 64	22.0	–	78.0
Amoxicillin-clavulanate	8	32	≤ 0.12– ≥ 64	56.0	–	44.0
Piperacillin-tazobactam	2	32	0.25– ≥ 256	84.9	4.4	10.7
Aztreonam	0.12	1	≤ 0.015– ≥ 256	90.5	1.9	7.6
Imipenem	0.12	0.25	≤ 0.03– ≥ 16	99.5	0.1	0.4
Meropenem	0.03	0.06	0.008– ≥ 32	99.4	0.4	0.3
Colistin^b^ (N = 885)	0.25	1	≤ 0.06– ≥ 16	99.1	–	0.9
Amikacin	2	8	≤ 0.25– ≥ 128	94.4	–	5.6
Tigecycline	0.25	0.5	≤ 0.015–4	95.5	–	4.5

– Indicates no breakpoint for the agent

ESBL, extended-spectrum β-lactamase; %I, percentage of isolates susceptible, increased exposure; MIC, minimum inhibitory concentration; MIC_50_, MIC required to inhibit growth of 50% of isolates (mg/L); MIC_90_, MIC required to inhibit growth of 90% of isolates (mg/L); %R, percentage of isolates resistant; %S, percentage of isolates susceptible, standard dosing

^a^Not suitable for use in the treatment of infections caused by ESBL-positive isolates

^b^Colistin was included on the comparator panel from 2014 onwards

Susceptibility rates to amikacin among ESBL-negative isolates were similar in all regions, from 94.4% in Africa/Middle East and Latin America to 96.5% in Europe. Among ESBL-positive isolates, susceptibility to amikacin was lower (79.9% in Europe to 89.8% in Asia/South Pacific). The susceptibility rates observed among ESBL-negative isolates to piperacillin-tazobactam were lowest in Europe (79.6%) and highest in Latin America (84.9%). In comparison, rates of susceptibility to piperacillin-tazobactam among ESBL-positive isolates were lower in each region, ranging from 61.1 to 74.0%.

High rates of susceptibility were observed among ESBL-negative levofloxacin-resistant *E. coli* for cefepime in all regions (between 91.1 and 93.3%) and for ceftazidime in three of the four regions (91.0 to 93.0%). A lower susceptibility rate to ceftazidime of 77.9% was observed among ESBL-negative isolates in Asia/South Pacific. Few ESBL-positive isolates from any region were susceptible to cefepime or ceftazidime (≤ 6.3%). Susceptibility rates to ampicillin and amoxicillin-clavulanate were lower compared with all other agents in each region for ESBL-negative isolates. Among each regional set of ESBL-positive isolates, susceptibility rates to ampicillin and amoxicillin-clavulanate were ≤ 41.6%.

In vitro activity data, by year, for ceftazidime-avibactam, colistin, meropenem, imipenem, and tigecycline against ESBL-positive and ESBL-negative isolates are presented in Additional file 1: Tables S1–S5. Over time, ceftazidime-avibactam, colistin, meropenem and imipenem showed consistently high and stable rates of susceptibility (≥ 96.7%) in Africa/Middle East, Europe and Latin America (Additional file 1: Tables S1–S4). For ESBL-positive isolates collected in the Asia/South Pacific region, reduced susceptibility rates were observed in 2018 to ceftazidime-avibactam (91.8%, Additional file 1: Table S1), and to imipenem (90.4%) and meropenem (91.1%) (Additional file 1: Tables S3 and S4) when compared with each of the preceding years. Susceptibility to tigecycline was > 92.6% between 2013 and 2018; rates of susceptibility were lower in 2012.

### Regional trend tests against levofloxacin-resistant *E. coli* over time

**Fig. 1:**
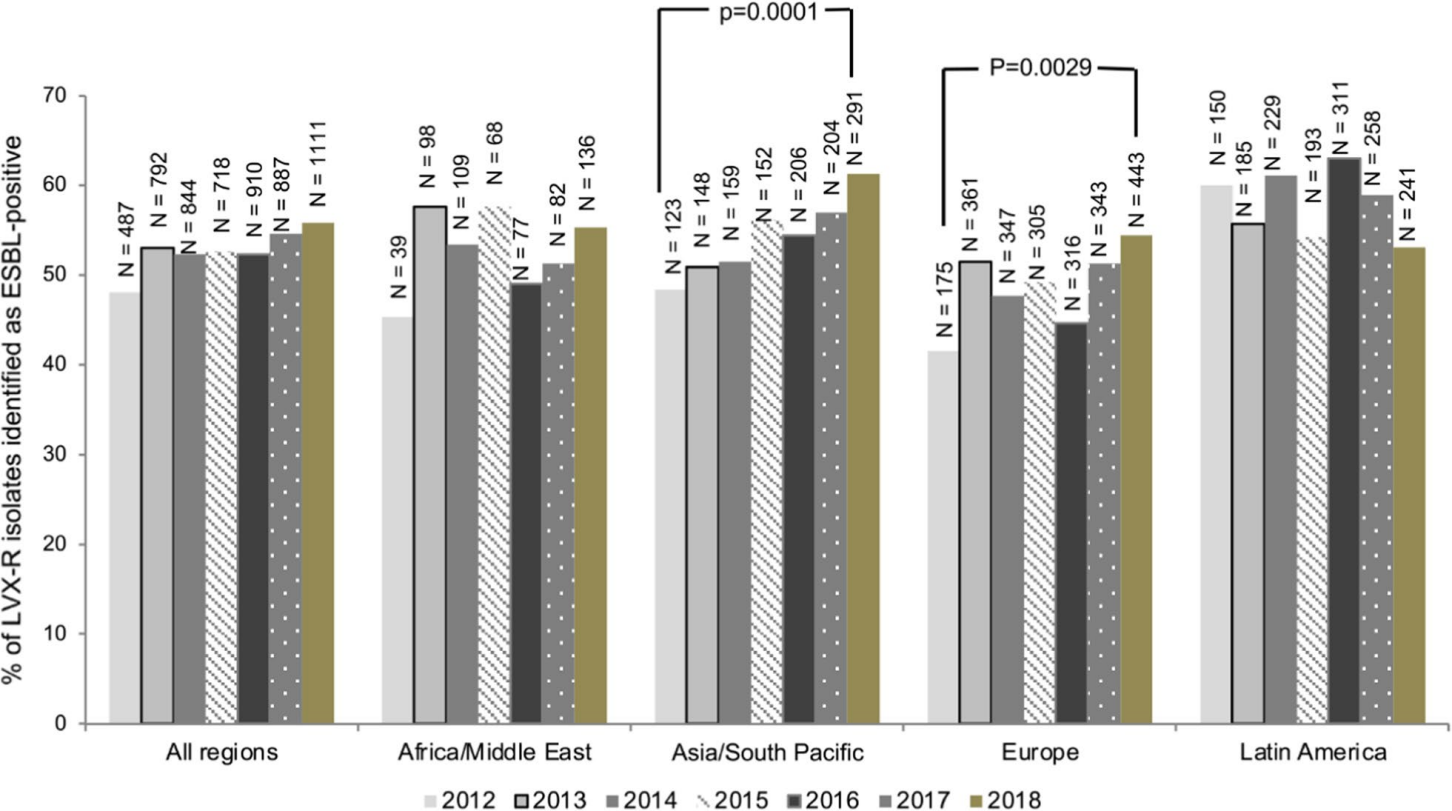
Proportion of levofloxacin-resistant *Escherichia coli* isolates identified as ESBL-positive, 2012–2018. **All regions:** 2012, n = 487/1012; 2013, n = 792/1494; 2014, n = 844/1615; 2015, n = 718/1365; 2016, n = 901/1739; 2017, n = 887/1625; 2018, n = 1111/1990. **Africa/Middle East:** 2012, n = 39/86; 2013, n = 98/170; 2014, n = 109/204; 2015, n = 68/118; 2016, n = 77/157; 2017, n = 82/160; 2018, n = 136/246. **Asia/South Pacific:** 2012, n = 123/254; 2013, n = 148/291; 2014, n = 159/309; 2015, n = 152/271; 2016, n = 206/379; 2017, n = 204/358; 2018, n = 291/475. **Europe:** 2012, n = 175/422; 2013, n = 361/701; 2014, n = 347/727; 2015, n = 305/620; 2016, n = 316/709; 2017, n = 343/669; 2018, n = 443/815. **Latin America:** 2012, n = 150/250; 2013, 185/332; 2014, n = 229/375; 2015, n = 193/356; 2016, n = 311/494; 2017, n = 258/438; 2018, n = 241/454

Figure 1 shows the proportion of levofloxacin-resistant *E. coli* isolates identified as ESBL-positive from each region and by year. Any changes in the rates of ESBL-positive, levofloxacin-resistant *E. coli* over time were not statistically significant in Africa/Middle East and Latin America. For isolates from Europe and Asia/Pacific there was a statistically significant increase in the rates of ESBL-positive isolates over time (p = 0.0029 and p = 0.0001, respectively) with rates in 2018 of 54.4% in Europe and 61.3% in Asia–Pacific.

## Discussion

This analysis of levofloxacin-resistant *E. coli* isolates collected between 2012 and 2018 in four geographical regions as part of the ATLAS study, showed high susceptibility rates to ceftazidime-avibactam among ESBL-positive and ESBL-negative isolates. Data also showed high susceptibility rates to colistin, meropenem, imipenem, and tigecycline, rates that were common to all regions and both ESBL-positive and ESBL-negative isolates. Susceptibility to cefepime, ceftazidime and aztreonam was also high against ESBL-negative isolates; however, susceptibility was reduced against ESBL-positive isolates with < 10% of isolates susceptible to cefepime, ceftazidime or aztreonam.

The year-by-year analysis for the Asia/Pacific region revealed lower rates of susceptibility to meropenem, imipenem and ceftazidime-avibactam in 2018 when compared with the 2012–2017 period. There have been reports of *E. coli* strains that are resistant to fluoroquinolones becoming more widespread during recent years [23]. Of particular concern has been the global spread of *E. coli* strain ST131, which is characterised by co-resistance to fluoroquinolones and other agents [8–10, 24, 25]. It is unlikely that this strain could be the cause of the lowered susceptibility we observed to ceftazidime-avibactam and the two carbapenems in the Asia/South Pacific region in 2018. Among *E. coli* ST131 the rate of resistance to carbapenems is considered to be low, and a recent study of the in vitro activity of ceftazidime-avibactam and comparators against *E. coli* ST131 isolates in the USA reported no resistance to ceftazidime-avibactam or meropenem [26]. A possible explanation may be the appearance of metallo-β-lactamases (MBLs) in isolates collected in the Asia/South Pacific region during 2018. Whilst MBL-positive isolates are frequently reported among *Klebsiella pneumoniae*, they are also disseminated to a lesser extent among *E. coli*, and so their inclusion among a population of levofloxacin-resistant isolates would be plausible [27].

The ATLAS program is intended for antimicrobial surveillance and is not designed as an epidemiological study. Therefore, the observations regarding the frequency of ESBLs need to be considered with caution. Furthermore, it must be remembered that the isolates included in this analysis are all levofloxacin-resistant, predisposing the collection to higher rates of ESBL-positive isolates than might be identified in other clinical collections of *E. coli*. Additionally, centres that have participated in ATLAS have not been required to do so in each year, so the analysis of longitudinal data could be influenced by changes in the distribution of isolates over time. In our analyses, data were not available for India or China, and so our findings cannot reasonably be applied to these individual countries. Whilst centres from many countries have participated in this analysis, their geographical distribution has focussed around the four main regions included in this analysis and so the observations that we present may not be fully representative of global susceptibility trends.

## Conclusions

In conclusion, we report that the in vitro susceptibility to ceftazidime-avibactam among levofloxacin-resistant *E. coli* isolates, including ESBL-positive isolates, collected from four geographical regions between 2012 and 2018 was consistently high (≥ 97.0%). Susceptibility to the comparator agents colistin, tigecycline, imipenem and meropenem was also high (≥ 94.1%), whilst susceptibility to other agents on the panel was lower, particularly among ESBL-positive isolates. A modest reduction in susceptibility to imipenem, meropenem, and ceftazidime-avibactam in the Asia/South Pacific region in 2018 warrants continued antimicrobial surveillance. The identification of global and regional trends of antimicrobial resistance can help to guide appropriate treatment of infectious disease where *E. coli* is the suspected or confirmed causative organism.

## Supplementary Information

**Additional file 1: Table S1.** Activity of ceftazidime-avibactam against levofloxacin-resistant *E. coli*; ATLAS, by region and year, 2012–2018. **Table S2.** Activity of colistin against levofloxacin-resistant *E. coli*, ATLAS, by region and year, 2014*–2018. **Table S3.** Activity of imipenem against levofloxacin-resistant *E. coli* isolates, ATLAS, by region and year, 2012–2018. **Table S4.** Activity of meropenem against levofloxacin-resistant *E. coli* isolates, ATLAS, by region and year, 2012–2018. **Table S5.** Activity of tigecycline against levofloxacin-resistant *E. coli*, ATLAS, by region and year, 2012–2018.

## Data Availability

The datasets generated and/or analysed during the current study are available from the corresponding author on reasonable request. Data from the global ATLAS study can be accessed at https://atlas-surveillance.com.

## Abbreviations

ATLAS: Antimicrobial Testing Leadership and Surveillance
ESBL: Extended-spectrum β-lactamase
INFORM: International Network for Optimal Resistance Monitoring
MALDI-TOF: Matrix-assisted laser desorption ionization-time of flight mass spectrometry
MBL: Metallo-β-lactamase
MIC: Minimum inhibitory concentration
UTI: Urinary tract infection
