# Supplementary material for: Antimicrobial activity of ceftazidime-avibactam and comparators against levofloxacin-resistant Escherichia coli collected from four geographic regions, 2012–2018
Source: Ann Clin Microbiol Antimicrob. 2022 Mar 21;21:13. doi: 10.1186/s12941-022-00504-8 (PMC8939136; doi:10.1186/s12941-022-00504-8)
Supplement: Supplementary file 1 — Additional file 1: Table S1. Activity of ceftazidime-avibactam against levofloxacin-resistant E. coli; ATLAS, by region and year, 2012–2018. Table S2. Activity of colistin against levofloxacin-resistant E. coli, ATLAS, by region and year, 2014*–2018. Table S3. Activity of imipenem against levofloxacin-resistant E. coli isolates, ATLAS, by region and year, 2012–2018. Table S4. Activity of meropenem against levofloxacin-resistant E. coli isolates, ATLAS, by region and year, 2012–2018. Table S5. Activity of tigecycline against levofloxacin-resistant E. coli, ATLAS, by region and year, 2012–2018. [file 12941_2022_504_MOESM1_ESM.docx]

#### Table S1. Activity of ceftazidime-avibactam against levofloxacin-resistant *E. coli*; ATLAS, by region and year, 2012–2018.

| Region,  Year | N | MIC_50_ (mg/L) | MIC_90_ (mg/L) | Range (mg/L) | %S | %I | %R |
| --- | --- | --- | --- | --- | --- | --- | --- |
| **Africa/Middle East, ESBL-positive** | | | |  |  |  |  |
| 2012 | 39 | 0.25 | 1 | ≤0.015–4 | 100 | – | 0.0 |
| 2013 | 98 | 0.12 | 0.25 | 0.03–2 | 100 | – | 0.0 |
| 2014 | 109 | 0.12 | 0.5 | 0.03–2 | 100 | – | 0.0 |
| 2015 | 68 | 0.25 | 1 | 0.06–16 | 98.5 | – | 1.5 |
| 2016 | 77 | 0.25 | 0.5 | 0.03–4 | 100 | – | 0.0 |
| 2017 | 82 | 0.12 | 0.25 | ≤0.015–4 | 100 | – | 0.0 |
| 2018 | 136 | 0.25 | 1 | ≤0.015–≥256 | 99.3 | – | 0.7 |
| **Africa/Middle East, ESBL-negative** | | | |  |  |  |  |
| 2012 | 47 | 0.12 | 0.25 | ≤0.015–2 | 100 | – | 0.0 |
| 2013 | 72 | 0.12 | 0.25 | ≤0.015–≥256 | 98.6 | – | 1.4 |
| 2014 | 95 | 0.06 | 0.25 | ≤0.015–≥256 | 98.9 | – | 1.1 |
| 2015 | 50 | 0.12 | 0.25 | ≤0.015–2 | 100 | – | 0.0 |
| 2016 | 80 | 0.06 | 0.12 | ≤0.015–1 | 100 | – | 0.0 |
| 2017 | 78 | 0.06 | 0.12 | ≤0.015–4 | 100 | – | 0.0 |
| 2018 | 110 | 0.12 | 0.25 | ≤0.015–8 | 100 | – | 0.0 |
| **Asia/South Pacific, ESBL-positive** | | | |  |  |  |  |
| 2012 | 123 | 0.25 | 1 | ≤0.015–≥256 | 99.2 | – | 0.8 |
| 2013 | 148 | 0.12 | 0.5 | ≤0.015–≥256 | 99.3 | – | 0.7 |
| 2014 | 159 | 0.06 | 0.25 | ≤0.015–8 | 100 | – | 0.0 |
| 2015 | 152 | 0.12 | 0.5 | ≤0.015–4 | 100 | – | 0.0 |
| 2016 | 206 | 0.12 | 0.5 | ≤0.015–≥256 | 97.1 | – | 2.9 |
| 2017 | 204 | 0.12 | 0.25 | ≤0.015–≥256 | 97.1 | – | 2.9 |
| 2018 | 291 | 0.25 | 4 | ≤0.015–≥256 | 91.8 | – | 8.2 |
| **Asia/South Pacific, ESBL-negative** | | | |  |  |  |  |
| 2012 | 131 | 0.12 | 0.25 | ≤0.015–1 | 100 | – | 0.0 |
| 2013 | 143 | 0.12 | 0.25 | ≤0.015–0.5 | 100 | – | 0.0 |
| 2014 | 150 | 0.06 | 0.25 | ≤0.015–0.5 | 100 | – | 0.0 |
| 2015 | 119 | 0.12 | 0.25 | ≤0.015–≥256 | 99.2 | – | 0.8 |
| 2016 | 173 | 0.12 | 0.25 | ≤0.015–2 | 100 | – | 0.0 |
| 2017 | 154 | 0.06 | 0.25 | ≤0.015–4 | 100 | – | 0.0 |
| 2018 | 184 | 0.12 | 2 | ≤0.015–≥256 | 97.3 | – | 2.7 |
| **Europe, ESBL-positive** | | | |  |  |  |  |
| 2012 | 175 | 0.25 | 0.5 | ≤0.015–64 | 99.4 | – | 0.6 |
| 2013 | 361 | 0.12 | 0.5 | ≤0.015–4 | 100 | – | 0.0 |
| 2014 | 347 | 0.12 | 0.5 | ≤0.015–4 | 100 | – | 0.0 |
| 2015 | 305 | 0.25 | 0.5 | ≤0.015–≥256 | 99.3 | – | 0.7 |
| 2016 | 316 | 0.12 | 0.5 | ≤0.015–4 | 100 | – | 0.0 |
| 2017 | 343 | 0.12 | 0.25 | ≤0.015–≥256 | 99.7 | – | 0.3 |
| 2018 | 443 | 0.25 | 0.5 | ≤0.015–≥256 | 98.6 | – | 1.4 |
| **Europe, ESBL-negative** | | | |  |  |  |  |
| 2012 | 247 | 0.12 | 0.25 | ≤0.015–16 | 99.6 | – | 0.4 |
| 2013 | 340 | 0.12 | 0.25 | ≤0.015–64 | 99.7 | – | 0.3 |
| 2014 | 380 | 0.06 | 0.25 | ≤0.015–4 | 100 | – | 0.0 |
| 2015 | 315 | 0.12 | 0.25 | ≤0.015–64 | 99.7 | – | 0.3 |
| 2016 | 393 | 0.12 | 0.25 | ≤0.015–≥256 | 99.0 | – | 1.0 |
| 2017 | 326 | 0.06 | 0.12 | ≤0.015–≥256 | 99.7 | – | 0.3 |
| 2018 | 372 | 0.12 | 0.25 | ≤0.015–≥256 | 99.2 | – | 0.8 |
| **Latin America, ESBL-positive** | | | |  |  |  |  |
| 2012 | 150 | 0.25 | 0.5 | ≤0.015–4 | 100 | – | 0.0 |
| 2013 | 185 | 0.12 | 0.25 | ≤0.015–4 | 100 | – | 0.0 |
| 2014 | 229 | 0.12 | 0.5 | ≤0.015–8 | 100 | – | 0.0 |
| 2015 | 193 | 0.25 | 0.5 | ≤0.015–32 | 99.0 | – | 1.0 |
| 2016 | 311 | 0.12 | 0.5 | ≤0.015–4 | 100 | – | 0.0 |
| 2017 | 258 | 0.12 | 0.25 | ≤0.015–≥256 | 99.6 | – | 0.4 |
| 2018 | 241 | 0.12 | 0.5 | ≤0.015–≥256 | 99.2 | – | 0.8 |
| **Latin America, ESBL-negative** | | | |  |  |  |  |
| 2012 | 100 | 0.12 | 0.25 | ≤0.015–1 | 100 | – | 0.0 |
| 2013 | 147 | 0.12 | 0.25 | ≤0.015–1 | 100 | – | 0.0 |
| 2014 | 146 | 0.06 | 0.25 | ≤0.015–2 | 100 | – | 0.0 |
| 2015 | 163 | 0.12 | 0.25 | ≤0.015–2 | 100 | – | 0.0 |
| 2016 | 183 | 0.12 | 0.25 | ≤0.015–0.5 | 100 | – | 0.0 |
| 2017 | 180 | 0.06 | 0.12 | ≤0.015–0.5 | 100 | – | 0.0 |
| 2018 | 213 | 0.12 | 0.25 | ≤0.015–4 | 100 | – | 0.0 |

– Indicates no breakpoint for the agent
ESBL, extended-spectrum β-lactamase; %I, percentage of isolates susceptible, increased exposure; MIC, minimum inhibitory concentration; MIC_50_, MIC required to inhibit growth of 50% of isolates (mg/L); MIC_90_, MIC required to inhibit growth of 90% of isolates (mg/L); %R, percentage of isolates resistant; %S, percentage of isolates susceptible, standard dosing.

#### Table S2. Activity of colistin against levofloxacin-resistant *E. coli*, ATLAS, by region and year, 2014*–2018.

| **Region,  Year** | **N** | **MIC_50_ (mg/L)** | **MIC_90_ (mg/L)** | **Range (mg/L)** | **%S** | **%I** | **%R** |
| --- | --- | --- | --- | --- | --- | --- | --- |
| **Africa/Middle East, ESBL-positive** | | | | |  |  |  |
| 2014 | 109 | 0.5 | 1 | 0.25–4 | 99.1 | – | 0.9 |
| 2015 | 68 | 1 | 1 | 0.25–4 | 98.5 | – | 1.5 |
| 2016 | 77 | 0.25 | 0.5 | 0.12–8 | 98.7 | – | 1.3 |
| 2017 | 82 | 0.25 | 0.5 | 0.12–8 | 98.8 | – | 1.2 |
| 2018 | 136 | 0.25 | 0.5 | ≤0.06–1 | 100 | – | 0.0 |
| **Africa/Middle East, ESBL-negative** | | | | |  |  |  |
| 2014 | 95 | 0.5 | 1 | 0.25–4 | 98.9 | – | 1.1 |
| 2015 | 50 | 1 | 1 | 0.25–2 | 100 | – | 0.0 |
| 2016 | 80 | 0.25 | 0.5 | ≤0.06–4 | 98.8 | – | 1.3 |
| 2017 | 78 | 0.25 | 0.5 | 0.12–≥16 | 98.7 | – | 1.3 |
| 2018 | 110 | 0.5 | 1 | ≤0.06–4 | 97.3 | – | 2.7 |
| **Asia/South Pacific, ESBL-positive** | | | |  |  |  |  |
| 2014 | 159 | 0.5 | 1 | 0.25–4 | 98.7 | – | 1.3 |
| 2015 | 152 | 0.25 | 1 | 0.25–4 | 98.7 | – | 1.3 |
| 2016 | 206 | 0.25 | 0.5 | 0.12–8 | 99.0 | – | 1.0 |
| 2017 | 204 | 0.25 | 0.5 | 0.12–8 | 98.5 | – | 1.5 |
| 2018 | 291 | 0.25 | 0.5 | ≤0.06–≥16 | 99.0 | – | 1.0 |
| **Asia/South Pacific, ESBL-negative** | | | |  |  |  |  |
| 2014 | 150 | 0.5 | 1 | 0.12–4 | 99.3 | – | 0.7 |
| 2015 | 119 | 0.5 | 1 | 0.12–4 | 99.2 | – | 0.8 |
| 2016 | 173 | 0.25 | 0.5 | ≤0.06–2 | 100 | – | 0.0 |
| 2017 | 154 | 0.25 | 0.5 | 0.12–1 | 100 | – | 0.0 |
| 2018 | 184 | 0.25 | 0.5 | ≤0.06–≥16 | 98.4 | – | 1.6 |
| **Europe, ESBL-positive** | | | |  |  |  |  |
| 2014 | 347 | 0.5 | 1 | 0.25–8 | 98.8 | – | 1.2 |
| 2015 | 305 | 0.5 | 1 | 0.12–≥16 | 99.3 | – | 0.7 |
| 2016 | 316 | 0.25 | 0.5 | ≤0.06–8 | 99.1 | – | 0.9 |
| 2017 | 343 | 0.25 | 0.5 | 0.12–8 | 99.4 | – | 0.6 |
| 2018 | 443 | 0.5 | 0.5 | ≤0.06–≥16 | 99.1 | – | 0.9 |
| **Europe, ESBL-negative** | | | |  |  |  |  |
| 2014 | 380 | 0.5 | 1 | 0.12–8 | 98.7 | – | 1.3 |
| 2015 | 315 | 0.5 | 1 | 0.12–8 | 99.4 | – | 0.6 |
| 2016 | 393 | 0.25 | 0.5 | ≤0.06–≥16 | 99.5 | – | 0.5 |
| 2017 | 326 | 0.25 | 0.5 | ≤0.06–≥16 | 98.5 | – | 1.5 |
| 2018 | 372 | 0.25 | 0.5 | ≤0.06–≥16 | 98.4 | – | 1.6 |
| **Latin America, ESBL-positive** | | | |  |  |  |  |
| 2014 | 229 | 0.5 | 1 | 0.25–2 | 100 | – | 0.0 |
| 2015 | 193 | 0.5 | 1 | 0.12–8 | 99.5 | – | 0.5 |
| 2016 | 311 | 0.25 | 0.5 | ≤0.06–8 | 99.4 | – | 0.6 |
| 2017 | 258 | 0.25 | 0.5 | 0.12–8 | 97.7 | – | 2.3 |
| 2018 | 241 | 0.25 | 0.5 | ≤0.06–8 | 99.2 | – | 0.8 |
| **Latin America, ESBL-negative** | | | |  |  |  |  |
| 2014 | 146 | 0.5 | 1 | 0.25–4 | 99.3 | – | 0.7 |
| 2015 | 163 | 0.5 | 1 | 0.12–4 | 98.2 | – | 1.8 |
| 2016 | 183 | 0.25 | 0.5 | 0.12–8 | 99.5 | – | 0.5 |
| 2017 | 180 | 0.25 | 0.5 | 0.12–≥16 | 99.4 | – | 0.6 |
| 2018 | 213 | 0.25 | 0.5 | ≤0.06–≥16 | 99.1 | – | 0.9 |

* Colistin was included on the comparator panel from 2014 onwards
– Indicates no breakpoint for the agent
ESBL, extended-spectrum β-lactamase; %I, percentage of isolates susceptible, increased exposure; MIC, minimum inhibitory concentration; MIC_50_, MIC required to inhibit growth of 50% of isolates (mg/L); MIC_90_, MIC required to inhibit growth of 90% of isolates (mg/L); %R, percentage of isolates resistant; %S, percentage of isolates susceptible, standard dosing.

#### Table S3. Activity of imipenem against levofloxacin-resistant *E. coli* isolates, ATLAS, by region and year, 2012–2018.

| Region, Year | N | MIC_50_ (mg/L) | MIC_90_ (mg/L) | Range (mg/L) | %S | %I | %R |
| --- | --- | --- | --- | --- | --- | --- | --- |
| **Africa/Middle East, ESBL-positive** | | | |  |  |  |  |
| 2012 | 39 | 0.12 | 0.5 | 0.06–0.5 | 100 | 0.0 | 0.0 |
| 2013 | 98 | 0.12 | 0.25 | 0.06–2 | 100 | 0.0 | 0.0 |
| 2014 | 109 | 0.25 | 0.25 | 0.12–1 | 100 | 0.0 | 0.0 |
| 2015 | 68 | 0.25 | 0.25 | 0.12–2 | 100 | 0.0 | 0.0 |
| 2016 | 77 | 0.25 | 0.5 | 0.12–2 | 100 | 0.0 | 0.0 |
| 2017 | 82 | 0.12 | 0.25 | 0.06–4 | 98.8 | 1.2 | 0.0 |
| 2018 | 136 | 0.25 | 0.25 | 0.12–≥16 | 97.8 | 0.7 | 1.5 |
| **Africa/Middle East, ESBL-negative** | | | |  |  |  |  |
| 2012 | 47 | 0.12 | 0.25 | 0.06–1 | 100 | 0.0 | 0.0 |
| 2013 | 72 | 0.12 | 0.25 | 0.06–8 | 98.6 | 0.0 | 1.4 |
| 2014 | 95 | 0.25 | 0.5 | 0.12–≥16 | 96.8 | 1.1 | 2.1 |
| 2015 | 50 | 0.12 | 0.25 | 0.12–1 | 100 | 0.0 | 0.0 |
| 2016 | 80 | 0.25 | 0.25 | 0.06–1 | 100 | 0.0 | 0.0 |
| 2017 | 78 | 0.12 | 0.25 | 0.06–2 | 100 | 0.0 | 0.0 |
| 2018 | 110 | 0.25 | 0.25 | 0.06–4 | 99.1 | 0.9 | 0.0 |
| **Asia/South Pacific, ESBL-positive** | | |  |  |  |  |  |
| 2012 | 123 | 0.12 | 0.5 | 0.06–≥16 | 99.2 | 0.0 | 0.8 |
| 2013 | 148 | 0.25 | 0.25 | ≤0.03–≥16 | 99.3 | 0.0 | 0.7 |
| 2014 | 159 | 0.25 | 0.25 | 0.06–2 | 100 | 0.0 | 0.0 |
| 2015 | 152 | 0.25 | 0.25 | 0.12–≥16 | 99.3 | 0.0 | 0.7 |
| 2016 | 206 | 0.25 | 0.5 | 0.06–≥16 | 96.6 | 0.0 | 3.4 |
| 2017 | 204 | 0.12 | 0.25 | 0.06–≥16 | 96.6 | 0.0 | 3.4 |
| 2018 | 291 | 0.25 | 2 | 0.06–≥16 | 90.4 | 1.4 | 8.2 |
| **Asia/South Pacific, ESBL-negative** | | |  |  |  |  |  |
| 2012 | 131 | 0.12 | 0.5 | 0.06–1 | 100 | 0.0 | 0.0 |
| 2013 | 143 | 0.12 | 0.25 | 0.06–2 | 100 | 0.0 | 0.0 |
| 2014 | 150 | 0.25 | 0.5 | 0.06–1 | 100 | 0.0 | 0.0 |
| 2015 | 119 | 0.25 | 0.5 | 0.12–≥16 | 99.2 | 0.0 | 0.8 |
| 2016 | 173 | 0.12 | 0.5 | 0.12–1 | 100 | 0.0 | 0.0 |
| 2017 | 154 | 0.12 | 0.5 | ≤0.03–4 | 99.4 | 0.6 | 0.0 |
| 2018 | 184 | 0.25 | 0.5 | 0.06–≥16 | 97.3 | 0.5 | 2.2 |
| **Europe, ESBL-positive** | | |  |  |  |  |  |
| 2012 | 175 | 0.12 | 0.25 | 0.06–≥16 | 98.9 | 0.0 | 1.1 |
| 2013 | 361 | 0.12 | 0.25 | ≤0.03–2 | 100 | 0.0 | 0.0 |
| 2014 | 347 | 0.25 | 0.5 | 0.06–4 | 99.7 | 0.3 | 0.0 |
| 2015 | 305 | 0.25 | 0.5 | 0.06–≥16 | 97.0 | 1.6 | 1.3 |
| 2016 | 316 | 0.12 | 0.25 | ≤0.03–4 | 99.1 | 0.9 | 0.0 |
| 2017 | 343 | 0.12 | 0.25 | 0.06–≥16 | 99.7 | 0.0 | 0.3 |
| 2018 | 443 | 0.25 | 0.25 | 0.12–≥16 | 98.4 | 0.2 | 1.4 |
| **Europe, ESBL-negative** | | |  |  |  |  |  |
| 2012 | 247 | 0.12 | 0.5 | ≤0.03–≥16 | 96.8 | 1.2 | 2.0 |
| 2013 | 340 | 0.12 | 0.25 | 0.06–4 | 99.7 | 0.3 | 0.0 |
| 2014 | 380 | 0.25 | 0.5 | 0.06–8 | 98.9 | 0.3 | 0.8 |
| 2015 | 315 | 0.25 | 0.5 | 0.06–≥16 | 98.1 | 1.3 | 0.6 |
| 2016 | 393 | 0.12 | 0.25 | ≤0.03–≥16 | 98.5 | 0.5 | 1.0 |
| 2017 | 326 | 0.12 | 0.25 | ≤0.03–4 | 99.7 | 0.3 | 0.0 |
| 2018 | 372 | 0.12 | 0.25 | 0.06–≥16 | 98.9 | 0.0 | 1.1 |
| **Latin America, ESBL-positive** | | |  |  |  |  |  |
| 2012 | 150 | 0.12 | 0.25 | 0.06–4 | 99.3 | 0.7 | 0.0 |
| 2013 | 185 | 0.12 | 0.25 | 0.06–2 | 100 | 0.0 | 0.0 |
| 2014 | 229 | 0.25 | 0.25 | 0.06–≥16 | 99.1 | 0.4 | 0.4 |
| 2015 | 193 | 0.25 | 0.25 | 0.12–8 | 99.0 | 0.5 | 0.5 |
| 2016 | 311 | 0.25 | 0.5 | 0.06–8 | 98.4 | 1.0 | 0.6 |
| 2017 | 258 | 0.12 | 0.25 | 0.06–≥16 | 99.6 | 0.0 | 0.4 |
| 2018 | 241 | 0.25 | 0.25 | 0.12–≥16 | 99.2 | 0.0 | 0.8 |
| **Latin America, ESBL-negative** | | |  |  |  |  |  |
| 2012 | 100 | 0.12 | 0.25 | 0.06–4 | 99.0 | 1.0 | 0.0 |
| 2013 | 147 | 0.12 | 0.25 | 0.06–8 | 99.3 | 0.0 | 0.7 |
| 2014 | 146 | 0.12 | 0.25 | 0.06–8 | 99.3 | 0.0 | 0.7 |
| 2015 | 163 | 0.25 | 0.25 | 0.06–8 | 99.4 | 0.0 | 0.6 |
| 2016 | 183 | 0.12 | 0.25 | ≤0.03–≥16 | 99.5 | 0.0 | 0.5 |
| 2017 | 180 | 0.12 | 0.25 | 0.06–2 | 100 | 0.0 | 0.0 |
| 2018 | 213 | 0.12 | 0.25 | 0.12–8 | 99.5 | 0.0 | 0.5 |

ESBL, extended-spectrum β-lactamase; %I, percentage of isolates susceptible, increased exposure; MIC, minimum inhibitory concentration; MIC_50_, MIC required to inhibit growth of 50% of isolates (mg/L); MIC_90_, MIC required to inhibit growth of 90% of isolates (mg/L); %R, percentage of isolates resistant; %S, percentage of isolates susceptible, standard dosing.

#### Table S4. Activity of meropenem against levofloxacin-resistant *E. coli* isolates, ATLAS, by region and year, 2012–2018.

| Region,  Year | N | MIC_50_ (mg/L) | MIC_90_ (mg/L) | Range (mg/L) | %S | %I | %R |
| --- | --- | --- | --- | --- | --- | --- | --- |
| **Africa/Middle East, ESBL-positive** | | |  |  |  |  |  |
| 2012 | 39 | 0.03 | 0.06 | 0.015–0.06 | 100 | 0.0 | 0.0 |
| 2013 | 98 | 0.03 | 0.06 | 0.015–4 | 99.0 | 1.0 | 0.0 |
| 2014 | 109 | 0.03 | 0.06 | 0.015–1 | 100 | 0.0 | 0.0 |
| 2015 | 68 | 0.06 | 0.12 | 0.03–0.25 | 100 | 0.0 | 0.0 |
| 2016 | 77 | 0.06 | 0.12 | 0.015–0.5 | 100 | 0.0 | 0.0 |
| 2017 | 82 | 0.03 | 0.03 | 0.015–0.5 | 100 | 0.0 | 0.0 |
| 2018 | 136 | 0.06 | 0.06 | 0.06–≥32 | 98.5 | 0.7 | 0.7 |
| **Africa/Middle East, ESBL-negative** | | | |  |  |  |  |
| 2012 | 47 | 0.03 | 0.03 | 0.015–0.06 | 100 | 0.0 | 0.0 |
| 2013 | 72 | 0.03 | 0.03 | 0.008–16 | 98.6 | 0.0 | 1.4 |
| 2014 | 95 | 0.03 | 0.06 | 0.015–16 | 96.8 | 1.1 | 2.1 |
| 2015 | 50 | 0.03 | 0.06 | 0.015–0.25 | 100 | 0.0 | 0.0 |
| 2016 | 80 | 0.03 | 0.06 | 0.015–0.25 | 100 | 0.0 | 0.0 |
| 2017 | 78 | 0.015 | 0.03 | 0.015–1 | 100 | 0.0 | 0.0 |
| 2018 | 110 | 0.06 | 0.06 | 0.06–1 | 100 | 0.0 | 0.0 |
| **Asia/South Pacific, ESBL-positive** | | |  |  |  |  |  |
| 2012 | 123 | 0.03 | 0.12 | 0.015–16 | 98.4 | 0.0 | 1.6 |
| 2013 | 148 | 0.03 | 0.06 | 0.015–16 | 99.3 | 0.0 | 0.7 |
| 2014 | 159 | 0.03 | 0.06 | 0.015–0.12 | 100 | 0.0 | 0.0 |
| 2015 | 152 | 0.03 | 0.06 | 0.015–16 | 98.7 | 0.7 | 0.7 |
| 2016 | 206 | 0.03 | 0.12 | 0.008–16 | 96.6 | 1.0 | 2.4 |
| 2017 | 204 | 0.03 | 0.06 | 0.015–16 | 96.6 | 1.0 | 2.5 |
| 2018 | 291 | 0.06 | 1 | 0.06–≥32 | 91.1 | 0.3 | 8.6 |
| **Asia/South Pacific, ESBL-negative** | | |  |  |  |  |  |
| 2012 | 131 | 0.03 | 0.06 | 0.015–0.25 | 100 | 0.0 | 0.0 |
| 2013 | 143 | 0.03 | 0.03 | 0.015–0.06 | 100 | 0.0 | 0.0 |
| 2014 | 150 | 0.03 | 0.06 | ≤0.004–0.25 | 100 | 0.0 | 0.0 |
| 2015 | 119 | 0.03 | 0.06 | 0.015–16 | 99.2 | 0.0 | 0.8 |
| 2016 | 173 | 0.03 | 0.06 | 0.015–0.25 | 100 | 0.0 | 0.0 |
| 2017 | 154 | 0.015 | 0.03 | 0.008–2 | 100 | 0.0 | 0.0 |
| 2018 | 184 | 0.06 | 0.12 | 0.06–≥32 | 97.3 | 0.5 | 2.2 |
| **Europe, ESBL-positive** | | |  |  |  |  |  |
| 2012 | 175 | 0.03 | 0.06 | 0.008–4 | 99.4 | 0.6 | 0.0 |
| 2013 | 361 | 0.03 | 0.06 | ≤0.004–0.25 | 100 | 0.0 | 0.0 |
| 2014 | 347 | 0.03 | 0.06 | 0.015–8 | 99.7 | 0.3 | 0.0 |
| 2015 | 305 | 0.03 | 0.12 | 0.008–16 | 96.7 | 2.6 | 0.7 |
| 2016 | 316 | 0.03 | 0.06 | 0.015–8 | 99.7 | 0.3 | 0.0 |
| 2017 | 343 | 0.03 | 0.03 | 0.008–16 | 99.4 | 0.3 | 0.3 |
| 2018 | 443 | 0.06 | 0.12 | 0.06–≥32 | 97.7 | 0.5 | 1.8 |
| **Europe, ESBL-negative** | | |  |  |  |  |  |
| 2012 | 247 | 0.03 | 0.06 | ≤0.004–16 | 99.6 | 0.0 | 0.4 |
| 2013 | 340 | 0.03 | 0.03 | ≤0.004–16 | 99.7 | 0.0 | 0.3 |
| 2014 | 380 | 0.03 | 0.06 | 0.008–8 | 99.2 | 0.8 | 0.0 |
| 2015 | 315 | 0.03 | 0.06 | 0.008–16 | 99.0 | 0.6 | 0.3 |
| 2016 | 393 | 0.03 | 0.06 | 0.008–16 | 98.5 | 0.8 | 0.8 |
| 2017 | 326 | 0.015 | 0.03 | 0.008–4 | 99.7 | 0.3 | 0.0 |
| 2018 | 372 | 0.06 | 0.06 | 0.06–≥32 | 98.7 | 0.3 | 1.1 |
| **Latin America, ESBL-positive** | | |  |  |  |  |  |
| 2012 | 150 | 0.03 | 0.06 | 0.015–8 | 99.3 | 0.7 | 0.0 |
| 2013 | 185 | 0.03 | 0.06 | 0.015–4 | 99.5 | 0.5 | 0.0 |
| 2014 | 229 | 0.03 | 0.06 | 0.015–16 | 98.7 | 0.4 | 0.9 |
| 2015 | 193 | 0.03 | 0.06 | 0.015–16 | 99.0 | 0.5 | 0.5 |
| 2016 | 311 | 0.03 | 0.06 | 0.015–8 | 98.7 | 1.3 | 0.0 |
| 2017 | 258 | 0.03 | 0.03 | 0.015–16 | 98.8 | 0.8 | 0.4 |
| 2018 | 241 | 0.06 | 0.06 | 0.06–≥32 | 99.2 | 0.0 | 0.8 |
| **Latin America, ESBL-negative** | | |  |  |  |  |  |
| 2012 | 100 | 0.03 | 0.03 | 0.008–4 | 99.0 | 1.0 | 0.0 |
| 2013 | 147 | 0.03 | 0.03 | 0.008–4 | 99.3 | 0.7 | 0.0 |
| 2014 | 146 | 0.03 | 0.06 | 0.015–8 | 99.3 | 0.7 | 0.0 |
| 2015 | 163 | 0.03 | 0.06 | 0.008–8 | 99.4 | 0.6 | 0.0 |
| 2016 | 183 | 0.03 | 0.06 | 0.015–16 | 99.5 | 0.0 | 0.5 |
| 2017 | 180 | 0.015 | 0.03 | 0.008–0.12 | 100 | 0.0 | 0.0 |
| 2018 | 213 | 0.06 | 0.06 | 0.06–≥32 | 99.1 | 0.0 | 0.9 |

ESBL, extended-spectrum β-lactamase; %I, percentage of isolates susceptible, increased exposure; MIC, minimum inhibitory concentration; MIC_50_, MIC required to inhibit growth of 50% of isolates (mg/L); MIC_90_, MIC required to inhibit growth of 90% of isolates (mg/L); %R, percentage of isolates resistant; %S, percentage of isolates susceptible, standard dosing.

#### Table S5. Activity of tigecycline against levofloxacin-resistant *E. coli*, ATLAS, by region and year, 2012–2018.

| Region,  Year | N | MIC_50_ (mg/L) | MIC_90_ (mg/L) | Range (mg/L) | %S | %I | %R |
| --- | --- | --- | --- | --- | --- | --- | --- |
| **Africa/Middle East, ESBL-positive** | | |  |  |  |  |  |
| 2012 | 39 | 0.5 | 1 | 0.12–4 | 84.6 | – | 15.4 |
| 2013 | 98 | 0.25 | 0.5 | 0.06–2 | 92.9 | – | 7.1 |
| 2014 | 109 | 0.25 | 0.5 | 0.06–1 | 96.3 | – | 3.7 |
| 2015 | 68 | 0.25 | 0.5 | 0.06–1 | 98.5 | – | 1.5 |
| 2016 | 77 | 0.12 | 0.5 | 0.12–0.5 | 100 | – | 0.0 |
| 2017 | 82 | 0.25 | 0.5 | 0.06–4 | 98.8 | – | 1.2 |
| 2018 | 136 | 0.25 | 0.5 | 0.06–1 | 97.1 | – | 2.9 |
| **Africa/Middle East, ESBL-negative** | | |  |  |  |  |  |
| 2012 | 47 | 0.5 | 1 | 0.12–2 | 80.9 | – | 19.1 |
| 2013 | 72 | 0.25 | 0.5 | 0.06–1 | 93.1 | – | 6.9 |
| 2014 | 95 | 0.25 | 0.5 | 0.12–1 | 96.8 | – | 3.2 |
| 2015 | 50 | 0.25 | 0.5 | 0.12–2 | 96.0 | – | 4.0 |
| 2016 | 80 | 0.25 | 0.5 | 0.06–4 | 98.8 | – | 1.3 |
| 2017 | 78 | 0.25 | 0.5 | 0.06–4 | 93.6 | – | 6.4 |
| 2018 | 110 | 0.25 | 0.5 | 0.06–1 | 98.2 | – | 1.8 |
| **Asia/South Pacific, ESBL-positive** | | |  |  |  |  |  |
| 2012 | 123 | 0.25 | 1 | 0.12–2 | 87.8 | – | 12.2 |
| 2013 | 148 | 0.25 | 0.5 | 0.06–4 | 92.6 | – | 7.4 |
| 2014 | 159 | 0.25 | 0.5 | 0.06–2 | 94.3 | – | 5.7 |
| 2015 | 152 | 0.25 | 0.5 | 0.06–1 | 97.4 | – | 2.6 |
| 2016 | 206 | 0.25 | 0.5 | 0.03–≥16 | 96.6 | – | 3.4 |
| 2017 | 204 | 0.25 | 0.5 | 0.03–8 | 95.6 | – | 4.4 |
| 2018 | 291 | 0.25 | 0.5 | 0.06–≥16 | 93.8 | – | 6.2 |
| **Asia/South Pacific, ESBL-negative** | | |  |  |  |  |  |
| 2012 | 131 | 0.25 | 1 | 0.06–2 | 87.8 | – | 12.2 |
| 2013 | 143 | 0.25 | 0.5 | 0.06–2 | 95.8 | – | 4.2 |
| 2014 | 150 | 0.25 | 0.5 | 0.03–2 | 94.0 | – | 6.0 |
| 2015 | 119 | 0.25 | 0.5 | 0.06–2 | 90.8 | – | 9.2 |
| 2016 | 173 | 0.25 | 0.25 | 0.06–1 | 99.4 | – | 0.6 |
| 2017 | 154 | 0.25 | 0.5 | 0.06–4 | 95.5 | – | 4.5 |
| 2018 | 184 | 0.25 | 0.25 | 0.06–2 | 96.7 | – | 3.3 |
| **Europe, ESBL-positive** | | |  |  |  |  |  |
| 2012 | 175 | 0.5 | 1 | 0.03–4 | 84.6 | – | 15.4 |
| 2013 | 361 | 0.25 | 0.5 | 0.06–2 | 95.3 | – | 4.7 |
| 2014 | 347 | 0.25 | 0.5 | 0.06–2 | 95.7 | – | 4.3 |
| 2015 | 305 | 0.25 | 0.5 | ≤0.015–2 | 96.4 | – | 3.6 |
| 2016 | 316 | 0.25 | 0.5 | 0.03–1 | 99.4 | – | 0.6 |
| 2017 | 343 | 0.25 | 0.5 | 0.06–4 | 98.5 | – | 1.5 |
| 2018 | 443 | 0.12 | 0.25 | 0.03–2 | 98.2 | – | 1.8 |
| **Europe, ESBL-negative** | | |  |  |  |  |  |
| 2012 | 247 | 0.5 | 1 | 0.12–4 | 78.9 | – | 21.1 |
| 2013 | 340 | 0.25 | 0.5 | 0.06–2 | 90.9 | – | 9.1 |
| 2014 | 380 | 0.25 | 0.5 | 0.03–4 | 94.5 | – | 5.5 |
| 2015 | 315 | 0.25 | 0.5 | 0.06–2 | 93.7 | – | 6.3 |
| 2016 | 393 | 0.25 | 0.5 | ≤0.015–1 | 99.0 | – | 1.0 |
| 2017 | 326 | 0.25 | 0.5 | 0.03–2 | 96.9 | – | 3.1 |
| 2018 | 372 | 0.12 | 0.25 | 0.03–2 | 99.2 | – | 0.8 |
| **Latin America, ESBL-positive** | | |  |  |  |  |  |
| 2012 | 150 | 0.5 | 1 | 0.12–4 | 85.3 | – | 14.7 |
| 2013 | 185 | 0.25 | 0.5 | ≤0.015–2 | 93.5 | – | 6.5 |
| 2014 | 229 | 0.25 | 0.5 | 0.06–4 | 96.1 | – | 3.9 |
| 2015 | 193 | 0.25 | 0.5 | 0.06–2 | 96.9 | – | 3.1 |
| 2016 | 311 | 0.25 | 0.5 | 0.06–1 | 98.7 | – | 1.3 |
| 2017 | 258 | 0.25 | 0.5 | 0.06–2 | 96.9 | – | 3.1 |
| 2018 | 241 | 0.25 | 0.5 | 0.03–2 | 97.9 | – | 2.1 |
| **Latin America, ESBL-negative** | | |  |  |  |  |  |
| 2012 | 100 | 0.25 | 1 | 0.06–2 | 88.0 | – | 12.0 |
| 2013 | 147 | 0.25 | 0.5 | ≤0.015–2 | 93.9 | – | 6.1 |
| 2014 | 146 | 0.25 | 0.5 | 0.12–2 | 97.3 | – | 2.7 |
| 2015 | 163 | 0.25 | 0.5 | 0.12–2 | 95.1 | – | 4.9 |
| 2016 | 183 | 0.25 | 0.5 | 0.06–4 | 97.8 | – | 2.2 |
| 2017 | 180 | 0.25 | 0.5 | ≤0.015–2 | 96.7 | – | 3.3 |
| 2018 | 213 | 0.12 | 0.25 | 0.03–2 | 96.2 | – | 3.8 |

– Indicates no breakpoint for the agent
ESBL, extended-spectrum β-lactamase; %I, percentage of isolates susceptible, increased exposure; MIC, minimum inhibitory concentration; MIC_50_, MIC required to inhibit growth of 50% of isolates (mg/L); MIC_90_, MIC required to inhibit growth of 90% of isolates (mg/L); %R, percentage of isolates resistant; %S, percentage of isolates susceptible, standard dosing.
